# Unraveling the Mechanism of Cork Spot-like Physiological Disorders in ‘Kurenainoyume’ Apples Based on Occurrence Location

**DOI:** 10.3390/plants13030381

**Published:** 2024-01-27

**Authors:** Eichi Imura, Mitsuho Nakagomi, Taishi Hayashida, Tomomichi Fujita, Saki Sato, Kazuhiro Matsumoto

**Affiliations:** 1 Faculty of Agriculture, Shizuoka University, Shizuoka 422-8529, Shizuoka, Japan; eichi_imura@aomori-itc.or.jp (E.I.); birodo.hikari.2@gmail.com (M.N.); 2 Apple Research Institute, Aomori Prefectural Industrial Technology Research Center, Kuroishi 036-0332, Aomori, Japan; 3 Fujisaki Farm, Faculty of Agriculture and Life Science, Hirosaki University, Fujisaki 038-3802, Aomori, Japan; hayashida@hirosaki-u.ac.jp (T.H.); fujita@hirosaki-u.ac.jp (T.F.); sa110006@hirosaki-u.ac.jp (S.S.)

**Keywords:** bagging, bitter pit, cork spot, computed tomography scanning, Jonathan spot, lenticel, red-fleshed apple, sunshine duration, vascular bundle, water deficit

## Abstract

Cork spot-like physiological disorder (CSPD) is a newly identified issue in ‘Kurenainoyume’ apples, yet its mechanism remains unclear. To investigate CSPD, we conducted morphological observations on ‘Kurenainoyume’ apples with and without pre-harvest fruit-bagging treatment using light-impermeable paper bags. Non-bagged fruit developed CSPD in mid-August, while no CSPD symptoms were observed in bagged fruit. The bagging treatment significantly reduced the proportion of opened lenticels, with only 17.9% in bagged fruit compared to 52.0% in non-bagged fruits. In non-bagged fruit, CSPD spots tended to increase from the lenticels, growing in size during fruit development. The cuticular thickness and cross-sectional area of fresh cells in CSPD spots were approximately 16 µm and 1600 µm², respectively. Healthy non-bagged fruit reached these values around 100 to 115 days after full bloom from mid- to late August. Microscopic and computerized tomography scanning observations revealed that many CSPD spots developed at the tips of vascular bundles. Therefore, CSPD initiation between opened lenticels and vascular bundle tips may be influenced by water stress, which is potentially caused by water loss, leading to cell death and the formation of CSPD spots.

## 1. Introduction

Apples (*Malus × domestica* Borkh.) are among the most popular deciduous fruit crops globally [1]. Traditional leading varieties, such as ‘Fuji’, ‘Delicious’, ‘Golden Delicious’, and ‘Gala’, typically exhibit white to yellowish flesh colors, although there may be variations in skin color [2]. In recent years, red-fleshed apple varieties, such as ‘Geneva’ and ‘Pink Pearl’, have been developed, but they face challenges for fresh consumption due to astringency and strong acidic properties, hindering their popularity [3]. However, newer red-fleshed varieties, such as ‘Baya Marisa’, ‘Rosette’ (UK), ‘Redlove’ (Switzerland), and ‘Weirouge’ (Germany and Italy), have gained commercial success due to their appealing characteristics, including an absence of astringency, sweetness, and enhanced nutritional values with higher anthocyanin content and antioxidant activities [4,5].

Japan has also seen the release of numerous red-fleshed apple varieties over the past quarter-century, such as ‘Rose Pearl’ and ‘Ruby Sweet’, from the National Agriculture and Food Research Organization [6,7], ‘Kurenainoyume’, ‘HFF60’, and ‘HFF33′ from Hirosaki University in Aomori Prefecture [8], and ‘Honeylouge’, ‘Columnar Rouge’, and ‘Red Sensation’ from Shinshu University in Nagano Prefecture [9,10]. The private apple grower/breeder Mr. Kazuo Yoshiie, in Nagano Prefecture, introduced red-fleshed varieties, such as ‘Nakano Shinku’, ‘Moon Rouge’, ‘Enbu’, and ‘Nakanono Kirameki’ [11]. Among these, ‘Kurenainoyume’, commercially released in 2010, is a pioneering red-fleshed apple cultivar in Japan, known for its sweetness, mild tartness, non-astringency, anthocyanin-rich content, and delectable taste, suitable for both raw and cooked consumption [8]. Categorized as a Type 2 red-fleshed variety, the red coloration of ‘Kurenainoyume’ is governed by the MYB family gene *MdMYB110a* [8].

The skin color of apples is controlled by the MYB family of genes, specifically *MdMYBA/1/10* [12]. In the Japanese market, consumers prefer Type 2 red-fleshed apples, such as ‘Kurenainoyume’, due to their attractive fruit size and taste compared to Type 1 red-fleshed varieties, such as ‘Maypole’, where the flesh coloration is controlled by the *MdMYBA/1/10* gene [13,14]. Consequently, the cultivation of Type 2 red-fleshed varieties has become prevalent in Japan. Despite its popularity, ‘Kurenainoyume’ has faced challenges, as growers have reported a unique spotted disorder on the fruit surface during development, a phenomenon previously unseen in global apple cultivation. This disorder, now termed cork spot-like physiological disorder (CSPD), is characterized by its lack of correlation with Ca and B content and its minimal development during cold storage. CSPD symptoms typically manifest in August, exhibiting some year-to-year variations and a strong correlation with sunshine duration from July to August, suggesting that prolonged sunshine during this period accelerates CSPD development [15]. Notably, shading has proven effective in reducing CSPD development, with double-layered paper bags providing nearly complete prevention from mid-July to late September [15,16].

CSPD occurrence and prevention using double-layered paper bags have been observed not only in ‘Kurenainoyume’ but also in other red-fleshed apple varieties, such as ‘HFF60′ and ‘HFF33′ [8]. These varieties share the same pollen parent, the ’Red-Fleshed Parent Strain A’, erroneously referred to as ‘Etter’s Gold’ in Japan. Consequently, CSPD is not exclusive to the ‘Kurenainoyume’ but is observed in red-fleshed apples more broadly. While paper bagging proves effective in preventing CSPD, its labor-intensive nature renders it unsuitable for the aging demographic of apple growers [17]. To develop more convenient methods for preventing CSPD, it is crucial to understand the mechanisms underlying its occurrence.

This study conducted four experiments to elucidate the CSPD mechanism, focusing on the place/position of CSPD spot occurrence. Firstly, we examined the effects of fruit growth speed and size on CSPD development. Subsequently, we revealed that CSPD developed from the opened lenticels on the fruit surface. Thirdly, we found that CSPD cells consisted of dead cells induced in August. Finally, we demonstrated that CSPD developed at the tip of the vascular bundles, using destructive sectional photographs and nondestructive computed tomography (CT) scans.

## 2. Results

### 2.1. Quality of Harvested Fruit and Time-Dependent Fruit Size Change

In this study, non-bagged fruit exhibited severe CSPD symptoms on the fruit surface, while bagging completely prevented this development (Table 1). Alongside CSPD development, non-bagged fruit had a slightly higher soluble solid content than bagged fruits. The growth trends of bagged and non-bagged fruit were very similar, indicating no remarkable differences between the two groups (Supplementary Figure S1).

### 2.2. CSPD Development and Its Relation to Fruit Size

**Figure 1:**
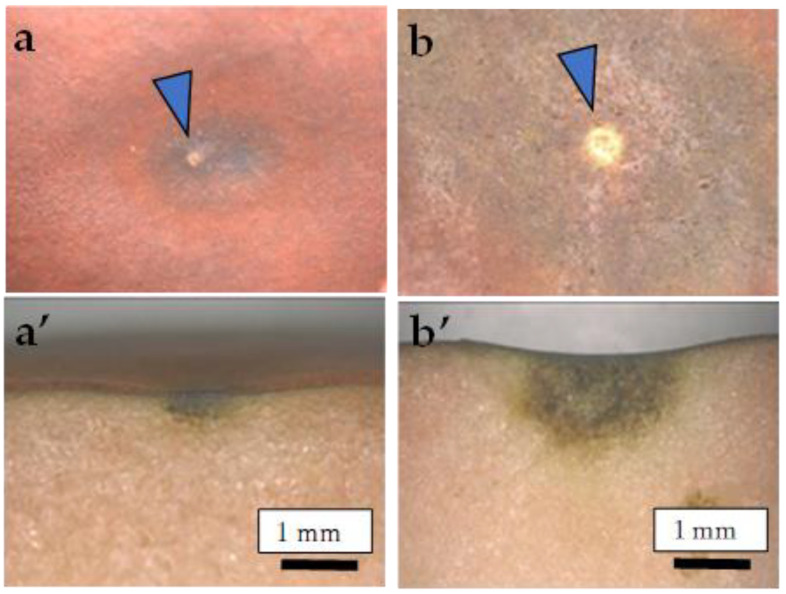
Typical symptoms of CSPD developed on the (**a**,**b**) fruit surface and (**a’**,**b’**) cross-section. (**a**,**a’**) <5 mm; (**b**,**b’**) 5–10 mm. Arrows indicate lenticels as the source of the symptoms.

CSPD began to develop from approximately 100 days after full bloom (DAFB) (mid-August) only on the non-bagged fruit surface in both years. There were no specific areas of CSPD development within the fruit surface, including the sunny (upper) and shady (lower) sides. However, the CSPD spots mainly originated from lenticels on the fruit surface (Figure 1). We examined the relationship between the number of CSPD spots and fruit fresh weight at harvest, obtaining an R^2^ value of 0.035 for the regression line coefficient of determination. Therefore, there was no significant relationship between the two factors (Supplementary Figure S2).

### 2.3. Effect of Fruit-Bagging Treatment on the Number, Size, and State of Lenticels

The fruit-bagging treatment did not affect the number of lenticels in the fruit. However, bagged fruit had smaller lenticels than non-bagged fruit. The proportion of opened lenticels decreased after the bagging treatment (Table 2).

### 2.4. Size Distribution of CSPD and the Dimensions of Cuticular and Cellular Components in the Affected Area

**Figure 2:**
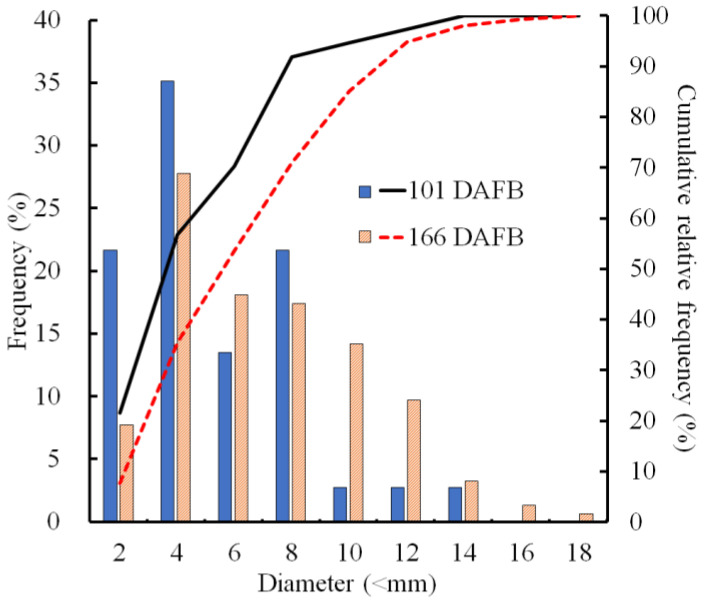
Size distribution of the cork spot-like physiological disorder (CSPD) developed in non-bagged ‘Kurenainoyume’ apple fruit. Bars indicate the frequency (%) of each diameter, and lines indicate the cumulative relative frequency (%). The blue filled bar and black line represent 101 DAFB (19 August 2019, n = 10 fruit), while the orange striped bar and red dot line represent 166 DAFB (23 October 2019, n = 20 fruit). On 101 and 166 DAFB, the CSPD numbers on the fruit surface were 3.5 ± 1.7 and 7.8 ± 1.8 (average per fruit ± SE), respectively.

The sizes of CSPD spots that developed on the non-bagged fruit surface were compared at two stages of fruit development. At 101 DAFB (mid-August), more than half of the spots were smaller than 4 mm, and spots larger than 10 mm constituted less than 10% of all the developed CSPD spots (Figure 2). At 166 DAFB (at harvest), the median diameter was approximately 6 mm, and large spots exceeding 10 mm were observed in more than 15% of the samples. Consequently, the cumulative relative frequency curves shifted towards larger sizes from 101 to 166 DAFB.

**Figure 3:**
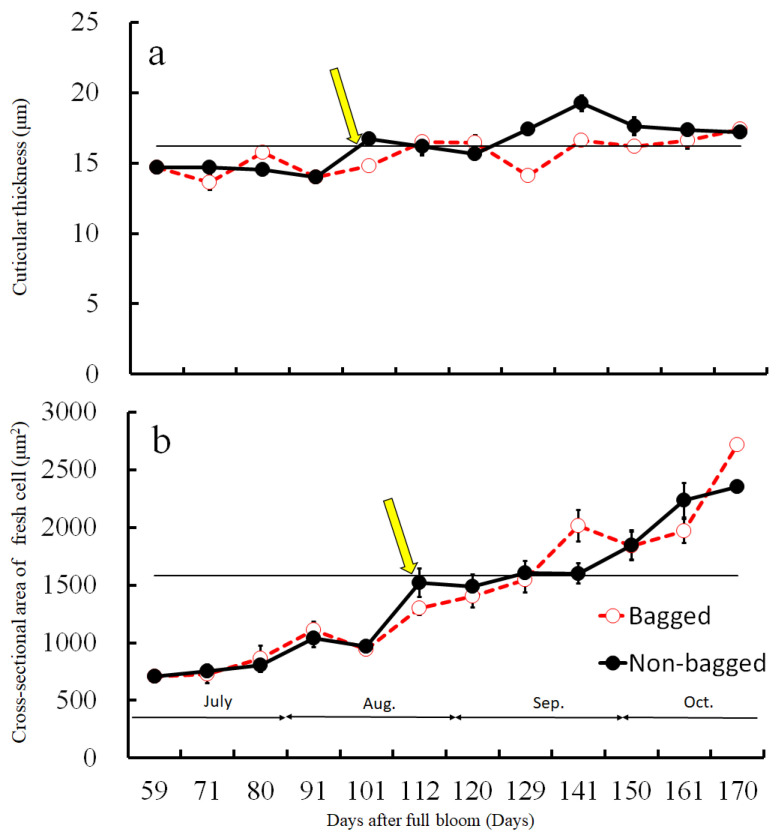
Changes in (**a**) cuticular thickness and (**b**) cross-sectional area of fresh cells during the fruit development duration of ‘Kurenainoyume’ apples, both paper-bagged and non-bagged in 2018. Vertical bars represent SE (n = 18). Horizontal lines with arrows indicate CSPD-affected portions. (**a**) Cuticular thickness; (**b**) Cross-sectional area of fresh cells at harvest of non-bagged ‘Kurenainoyume’ (23 October 2019, 166 DAFB).

**Figure 4:**
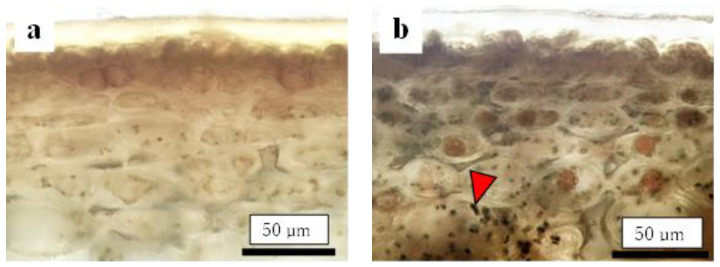
Comparison of cells between (**a**) healthy and (**b**) CSPD-affected areas of non-bagged ‘Kurenainoyume’ fruit stained with iodine. Slices were made from the same fruit. Black dots pointed out by arrows are starch grains.

Before assessing the cuticular thickness and cell size in the CSPD-affected areas, we examined the time-dependent changes in the size of healthy cuticular and flesh cells. The fruit-bagging treatment had no effect on cuticular thickness, although the bagged fruit exhibited slightly thinner cuticles at some time points (Figure 3a). The cross-sectional area of the fresh cells, indicating cell size, increased with time. However, the fruit-bagging treatment had no effect (Figure 3b). The cuticular thickness and cross-sectional area of fresh cells from the CSPD-affected portion at harvest are indicated by the horizontal line with arrows in Figure 3a,b, respectively. Values were approximately 16 µm and 1600 µm^2^, respectively. The non-bagged healthy fruit reached these values at approximately 100–115 DAFB from mid- to late August. Moreover, microscopic observation of iodine-stained slices of the CSPD-spot-developed portion confirmed the presence of many starch grains inside the CSPD-affected cells, although there were no starch grains in the healthy areas (Figure 4).

### 2.5. Destructive Observation of the Sliced Sections and the CSPD Spots of Non-Bagged ‘Kurenainoyume’ Fruit

**Figure 5:**
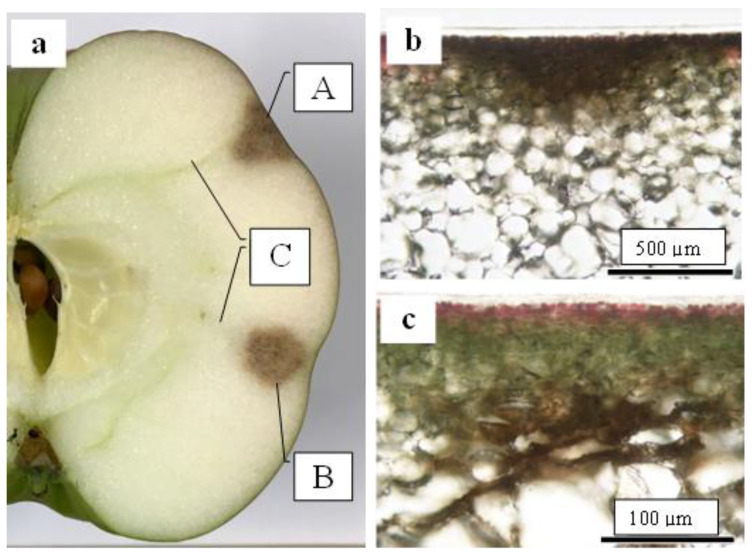
Two types of lesions typical of cork spot-like physiological disorders (CSPD) developed in non-bagged ‘Kurenainoyume’ apples. (**a**) Both types of CSPD developed in one fruit. (**A**) CSPD developed on the fruit surface; (**B**) CSPD developed inside the fruit; (**C**) vascular bundles; (**b**) CSPD developed on the fruit surface; (**c**) CSPD developed inside the fruit. The samples were harvested in (**a**): 10 October 2018, (**b**,**c**): 23 October 2019, respectively.

**Figure 6:**
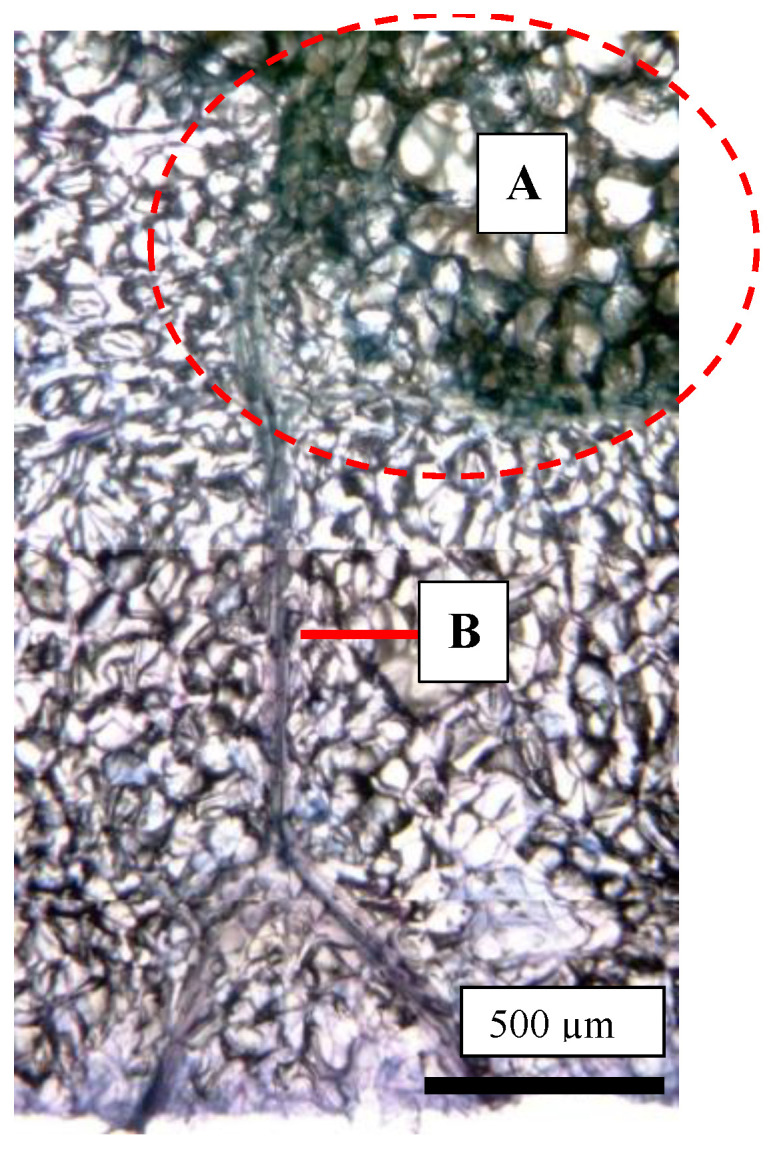
Trypan blue-stained photo of a cork spot-like physiological disorder (CSPD) lesion developed in non-bagged ‘Kurenainoyume’ apples. (**A**: the area enclosed by a red dotted circle) CSPD; (**B**) Vascular bundles. The fruit were harvested on 23 October 2019.

As shown in Figure 1, the CSPD spot is enlarged from the lenticel hole. However, in-depth observations of the sliced sections indicated two types of CSPD spots: one directly connected to the fruit surface (Figure 5a(A),b) and the other not (Figure 5a(B),c). Normally, the former type of CSPD spots was the majority, and the latter CSPD spots were very rare. However, in both types, spots were connected to vascular bundles inside the fruit flesh (Figure 5a(C)). Microscopic observations also indicated that many CSPD spots had developed at the tips of the vascular bundles (Figure 6).

### 2.6. Analysis of the Relationship between the CSPD Spots and the Vascular Bundles Using CT Scanning

**Figure 7:**
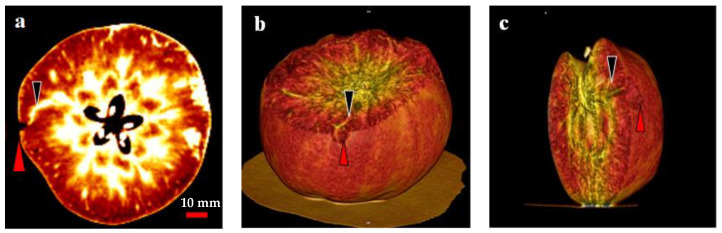
Computed tomography scanned images of a cork spot-like physiological disorder (CSPD) affected non-bagged ‘Kurenainoyume’ apple fruit. (**a**) 2-dimensional image; (**b**,**c**) 3-dimensional images. Red and black arrows indicate CSPD lesions and vascular bundles, respectively.

In the present study, two- and three-dimensional (2D and 3D) image analyses indicated a relationship between the CSPD lesions and vascular bundles (Figure 7). In the 2D image, air and water are distinguished by different colors: air is indicated in black, and water is indicated in white (Figure 7a). Therefore, severe CSPD symptoms on the fruit surface are indicated in black, and vascular bundles are indicated in white. As shown in Figure 7a, a higher density of vascular bundles was observed in the core region of the fruit, with some vascular bundles spreading from the core to the skin. We observed the spread of the vascular bundle, which was clearly connected to the CSPD spot. Except for this connection, there were no clear differences between bagged and non-bagged fruit.

Similar results were observed in the 3D images taken from the same area as the 2D image (Figure 7b,c). The dented surface is connected to a vascular bundle running away from the core region. Among the 10 analyzed disordered fruit, almost all severe CSPD lesions were connected to vascular bundles, regardless of whether the CSPD developed on the fruit surface or inside.

## 3. Discussion

CSPD development in the ‘Kurenainoyume’ apple fruit is a new type of physiological disorder that cannot be prevented by Ca and/or B application. However, it can be effectively controlled by preharvest fruit bagging using light-impermeable paper bags from mid-July to late September [15]. Unlike bitter pit symptoms [18], CSPD never develops during the post-harvest cold storage duration [19]; therefore, only the differential environmental conditions during more than 2 months of summer between bagged and non-bagged might determine whether CSPD develops.

In the present study, we considered the possibility that CSPD development in non-bagged fruit was accelerated by abnormal fruit growth. In Japanese apricots [20], it was reported that rapid fruit growth induces a physiological disorder called gamming syndrome. The susceptible cultivar ‘Benisashi’ exhibited explosive mesocarp cell elongation close to the endocarp during fruit development and created cavities filled with gum lumps. For the ‘Akizuki’ Japanese pear, Hayama et al. [21] reported that larger fruit accelerated the cork disorder development in the fruit. Reid and Kalcsits [22] reported that larger fruit tend to develop more severe symptoms than smaller fruit; therefore, as a practical technique, the cultivation of smaller fruit is useful for reducing the rate of bitter pit-suspected fruit. When we compared the fruit growth between bagged and non-bagged apples, we found no conspicuous differences in fruit development (Supplementary Figure S1). Nevertheless, CSPD development was reduced in bagged fruit, indicating the possibility that CSPD being induced by abnormal fruit development has declined.

Careful observation of the CSPD spots revealed that they initiated around the lenticels on the fruit surface (Figure 1). Lenticels are white to brown spots that develop on fruit surfaces [23]. Lenticels mainly develop from failed stomata and epidermal hair, as these sites do not undergo anticlinal division. Both sites conserve stress, and the strain causes rupture, leading to the development of a phelloderm. The phelloderm is filled with suberin, a polymer composed of hydroxy fatty acids, glycerol, and ferulic acid, acting as a site for water vapor permeance [24]. Normally, lenticel density differs among apple cultivars [24]. In the present study, the non-bagged fruit had a larger lenticel diameter than the bagged fruit (Table 2). Khanal et al. [24] reported that apple cultivars with a high density of lenticels and a higher total area of lenticels showed higher fruit transpiration. Maguire et al. [25] reported that approximately 20% of the total water loss from the apple’s surface was from lenticels; therefore, lenticels are the most common sites of water loss from the fruit [26]. Furthermore, Tomana [27] reported that “Jonathan spots”, a spot type developed by physiological disorders, were induced from the opened lenticels of the ‘Jonathan’ apples. Moreover, disordered fruits with opened lenticels showed a higher transpiration rate during storage because of the direct connection between the interior of the lenticels and the atmosphere. In our previous experiments using ‘Kurenainoyume’ fruit, no Jonathan spots symptoms were observed during storage regardless of the bagging treatment [19], as this physiological disorder was not solely induced by the reason for the opened lenticels. However, it could be valuable to point out that open lenticels are one of the most susceptible portions of fruit to drought stress. Endo [28] reported that bagging treatment using light-permeable paraffinized paper bags induced higher temperature and humidity conditions around Japanese pears than non-bagged ones. This may explain the reduction in CSPD spots in bagged fruit, where the bagging treatment may induce changes in the microenvironment of the ‘Kurenainoyume’ fruit surface and effectively prevent drought stress. In other words, non-bagged ‘Kurenainoyume’ may easily suffer from water stress, especially around the lenticels.

Our previous study showed that symptoms of CSPD in non-bagged ‘Kurenainoyume’ fruit are typically observed in August [15]. In the present study, we confirmed that CSPD was prominently observed in mid-August. The spot size increased until maturation in late October (Figure 2). Paper bagging from mid-July to late September completely prevented the development of CSPD (Table 1), as shown in previous reports [15]. Therefore, during this period, perhaps in August, the non-bagged ‘Kurenainoyume’ might have developed the incipient stage of the CSPD surface and/or inside the fruit. Microscopic observation of the fruit flesh that suffered from CSPD in mature non-bagged fruit indicated that the cell size was smaller than that of the healthy part of the flesh surrounding the damaged cells (Figure 3). In general, fruit flesh cells enlarge during growth via cell elongation [29]. ‘Kurenainoyume’ fruit flesh cells continuously enlarged during the growing season regardless of the fruit-bagging treatment (Figure 3b). Smaller cells in the CSPD-affected areas may indicate that cell enlargement in this portion stopped in the middle of the cell enlargement period until fruit maturation. The cell size in the CSPD-affected areas was almost the same as in the CSPD-unaffected areas in mid-to-late August (Figure 3). Starch grains remaining inside the CSPD-affected cells also indicated that cell activity was lost prior to sugar accumulation in the fruit (Figure 4).

There are several possibilities for the initiation of CSPD development from mid-August to late August. One possibility is the intensity of sunlight because light-impermeable paper-bagging treatment completely prevents CSPD development [15,16]. The sunshine duration from July to August is positively correlated with the rate of CSPD development [15]. However, when we checked the portion of CSPD development, spots were observed on the front surface of the Sun but also on the shaded surface. These results indicate that CSPD developed from open lenticels, strongly suggesting that the inducing factor is water stress. However, the CSPD spots were not always observed immediately below the fruit skin (Figure 5). In our study, some spots developed from deeper flesh areas that were a few millimeters inside the fruit’s surface. This result indicates that CSPD spots are initiated by water stress induced on the outer side of the fruit but also on the inner side. The fact that the CSPD spots were connected to vascular bundles (Figure 6 and Figure 7) can be used to reveal the mechanisms of CSPD development.

To assess the relationship between CSPD spots and vascular bundles non-destructively, we used an X-ray CT system in the present study. This system is primarily used for human medical testing. However, in recent years, the system has also been used to assess the interior quality of fruit cultivars, including apples [30], pears [31], and pomegranates [32]; this is because differences in water content among organs can be detected without additional agents [33,34]. Therefore, for apples, the vascular bundle distribution within the fruit was easily detected nondestructively, independent of the existence of physiological disorders in the water core [30]. We also found that the vascular bundles and CSPD were easily distinguishable from other fruit organs. Therefore, the vascular bundles contained more water than the other fruit parts, and the CSPD-affected area contained less water. The 3D analysis of the relationship between the vascular bundles and CSPD spots also showed that all CSPD spots were connected to the tips of the vascular bundles near the fruit’s surface (Figure 7). Thus, the CSPD-developed area is in an environment easily exposed to water stress through the vascular bundles.

Although we do not have a rigid answer as to why ‘Kurenainoyume’ apples easily suffer from CSPD in relation to water supplies, we can speculate that the ‘Kurenainoyume’ may be a water stress-susceptible cultivar. Further research is needed to reveal the water relationship between the plant body and the fruit of ‘Kurenainoyume’ apples.

## 4. Materials and Methods

### 4.1. Plant Materials

The apple cultivar ‘Kurenainoyume’ was utilized in the present study. ‘Kurenainoyume’ is a CSPD-susceptible cultivar whose outbreak can be mitigated by the paper bagging treatment. All apples were harvested at the Fujisaki Farm, Hirosaki University, Japan (40°39′25″ N, 140°29′9″ E) during the 2018 and 2019 seasons. The ‘Kurenainoyume’ was harvested from three trees, 12 years old (as of 2018), grafted onto *Malus prunifolia* trees and trained to a central leader form (5.0 × 3.5 m planting).

Approximately 30 randomly selected fruit per tree underwent a preharvest fruit-bagging treatment. The treated fruit were covered with light-impermeable, double-layered paper bags (two-layer bag, 194 × 162 mm; Masudaya Co., Aomori, Japan). The dates for full bloom, bagging, bag removal, and maturity for both cultivars over the 2 years are presented in Table 3. The remaining fruit were left uncovered using a paper bag (non-bagged fruit). Trees received routine annual horticultural care.

Starting from the beginning of June, three bagged and three unbagged fruit were randomly selected and harvested every 10 days until maturation. The harvested fruit were promptly transported to the experimental laboratory at Shizuoka University (Shizuoka, Japan) using a courier service at 4 °C for 36 h and used for subsequent experiments.

### 4.2. Fruit Development Analysis and Quality Measurement

For immature fruit, fresh weight, length, and diameter were measured using a digital caliper (A&D Company Ltd., Tokyo, Japan) and an electronic scale (OHAUS Corporation, NJ, USA), respectively. Additionally, for mature fruit, flesh firmness, soluble solid content, and titratable acidity were measured using the following procedures: flesh firmness was measured at two points on the equatorial area of the fruit without the skin using a penetrometer with an 11.1 mm tip (FT327; Facchini srl, Alfonsine, Italy). The total soluble solids content of the juice was determined using a digital refractometer (N-1; Atago, Tokyo, Japan). The total titratable acidity was measured by titration with 0.1 N NaOH and calculated as a malic acid equivalent.

### 4.3. Nondestructive Observation of Number and Structure of the CSPD

CSPD development in bagged and non-bagged fruit was checked periodically. We confirmed that the bagged fruit did not show any CSPD, whereas non-bagged fruit developed CSPD symptoms, similar to previous reports [15]. Therefore, during the initial stage of CSPD development (19 August 2019: 101 DAFB) and harvest day (23 October 2019: 166 DAFB), 10 and 20 non-bagged fruit were randomly selected from three ‘Kurenainoyume’ trees, respectively. The diameter of the CSPD symptom area was measured, and the size distribution of CSPD was analyzed. On harvest days, we analyzed the relationship between fruit fresh weight and the number of CSPD per fruit.

### 4.4. Observation of the Number and Structure of the Lenticels

In 2019, both non-bagged and bagged fruit were harvested on suitable days (23 October 2019: 166 DAFB), and the number and structure of the lenticels were observed. Five fruit were used for the observation. The number of lenticels was counted under an optical microscope (BH-2; Olympus Corporation, Tokyo, Japan) in three randomly selected visual fields (10 m^2^) per fruit (n = 15). The diameter of the lenticels was measured using randomly selected one or two lenticels per visual field on a crack scale (Niigata Seiki Co. Ltd., Niigata, Japan) (n = 25).

**Figure 8:**
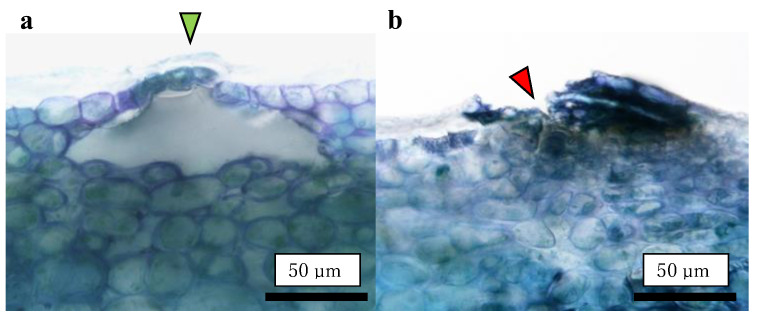
Typical images of lenticels. (**a**) Closed lenticel covered by the cuticular layer (green arrow); (**b**) Opened lenticel uncovered by the cuticular layer (red arrow). These images were captured using a non-bagged ‘Kurenainoyume’.

Destructive observations of the lenticels were conducted by making vertical slices of the tissues containing the lenticels. Vertical slices were prepared using a manual plant microtome (Nippon Medical & Chemical Instruments Co., Ltd., Osaka, Japan) with less than 50 μm thickness. A slice containing the center of the lenticel was selected and used for preparation. A distinction of opened or closed lenticel was made on an optical microscope equipped with a microscope camera (VEX12: Wraymer Inc., Osaka, Japan) (n = 25–30). An open lenticel was defined as one not closed by the cuticular layer, exposing fruit flesh cells directly to the external atmosphere (Figure 8).

### 4.5. Destructive Observation of CSPD, Cuticular Thickness, and Fresh Cell Size

Cuticular thickness and fresh cell size were measured in the harvested fruit used for fruit quality analysis in the 2018 season. Cubes approximately 10 mm in length that contained fruit skin were cut off from the CSPD-unaffected section of the fruit, according to the method described above. Six slices were randomly selected from each fruit, and the thickness of the cuticle layer and cross-sectional area of the flesh cells 5 mm under the cuticle layer were measured (n = 18).

Destructive observations of the CSPD areas were conducted using mature, non-bagged fruit. The preparations were prepared using the same methods used for the cuticular and cell size measurements. The cuticular thickness and cell size of the CSPD-affected areas were measured at the center of the disordered area (n = 18). Depending on the situation, disordered areas were stained with 0.01% toluidine blue O, 1.0% iodine solution, or 0.01% trypan blue. The dyeing times were 6, 5, and 0.5 min, respectively.

### 4.6. Nondestructive Image Scanning by CT

An X-ray CT system (Bright Speed Elite SD; GE Healthcare Japan Corporation, Tokyo, Japan) was used to investigate the relationship between CSPDs and fruit vascular bundles. The analysis was conducted at the Hamamatsu University School of Medicine, Hama-matsu, Shizuoka, Japan. An intact mature ‘Kurenainoyume’ fruit, which developed the CSPD, was set into a box, and sliced images were taken every 0.625 mm. For 2D image analysis, free imaging software (Weasis DICOM Medical Viewer v2.0.8, University Hospital of Geneva, Switzerland) was used. For 3D image analysis, a DICOM viewer (OsiriX v10, Newton Graphics, Inc., Hokkaido, Japan) was used to analyze changes in angles and colors. 

### 4.7. Statistical Analysis

Data were analyzed using the *t*-test, Tukey–Kramer honestly significant difference test, or Pearson’s chi-squared test using JMP 14 software v14.3.0 (SAS Institute, Cary, NC, USA), and significant differences between treatments were determined. Unless otherwise stated, differences were considered statistically significant at *p* < 0.05.

## 5. Conclusions

The CSPD in non-bagged ‘Kurenainoyume’ fruit is caused by cell death initiated in August because of external water stress through the opened lenticels and internal water stress through the vascular bundles. The apical portion of the vascular bundles just below the pericarp is exposed to severe water stress; therefore, cell death spreads around this portion, and cork spots are initiated. Preharvest fruit bagging using light-impermeable paper bags may maintain humidity around the fruit, thereby completely preventing CSPD development. Further research is required to confirm this hypothesis because the actual water status was not checked in this experiment. However, the identification of the morphological features related to CSPD development in this study will accelerate further research.

## Figures and Tables

**Table 1:** Fruit quality parameters of ‘Kurenainoyume’ apples harvested at a suitable maturation time (24 October 2018).

Treatment	CSPD	Fresh Weight (g)	Length (mm)	Diameter (mm)	Firmness (N)	Soluble Solid Contents (Brix°)	Titratable Acidity (%)
Non-bagged	Yes	302.1	73.2	92.1	72.2	11.3	0.87
Bagged	No	260.3	71.5	85.9	77.5	10.0	0.85
Significant		NS	NS	NS	NS	*	NS

* and NS indicate statistically significant and non-significant differences determined by *t*-test at *p* < 0.05 (n = 3), respectively.

**Table 2:** Effect of fruit-bagging treatment on the number of lenticels, diameter, and opened lenticels rate (%) of ‘Kurenainoyume’ apple at harvest (23 October 2019: 166 DAFB).

Treatment	Number of Lenticels (/10 mm^2^)	Diameter of Lenticels (mm)	Opened Lenticel Rate (%)
Non-bagged	4.5	0.50	52.0
Bagged	5.4	0.38	17.9
Significant	NS	*	**

*, **, and NS indicate a significant difference at the 5% and 1% levels, respectively, determined by *t*-test or Pearson’s chi-squared test (n = 15–25).

**Table 3:** Dates of full bloom, bagging, bag removing, and maturity of ‘Kurenainoyume’ apple in 2018 and 2019 grown in Fujisaki Farm, Hirosaki University, Japan.

Date	2018	2019
Full bloom	7 May	10 May
Bagging	6 July (59 *)	27 June (48)
Bag removal	25 September (141)	24 September (137)
Maturity	24 October (170)	23 October (166)

* Numbers inside parentheses indicate days after full bloom (DAFB).

## Data Availability

The data presented in this study are available upon request from the corresponding author. Please note that the data are not publicly available due to institutional policies.

## Supplementary Materials

The following supporting information can be downloaded at https://www.mdpi.com/article/10.3390/plants13030381/s1, Figure S1: Changes in (a) fruit fresh weight; (b) fruit length, and (c) fruit diameter during fruit development of both paper-bagged and non-bagged ‘Kurenainoyume’ apples in 2018; Figure S2: Relationship between the number of spots and fruit fresh weight of non-bagged ‘Kurenainoyume’ apples at harvest (23 October 2019, 166 DAFB) (n = 20).
