# Supplementary material for: Unraveling the Mechanism of Cork Spot-like Physiological Disorders in ‘Kurenainoyume’ Apples Based on Occurrence Location
Source: Plants (Basel). 2024 Jan 27;13(3):381. doi: 10.3390/plants13030381 (PMC10857259; doi:10.3390/plants13030381)
Supplement: Supplementary file 1 [file plants-13-00381-s001.zip › plants-2803430-supplementary.pdf]

Supplementary material

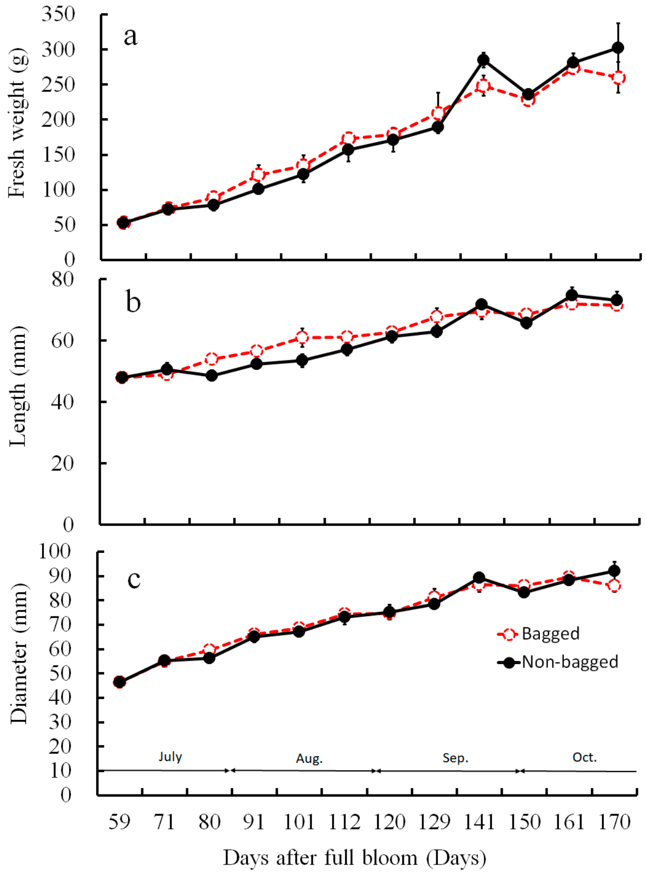

Figure S1. Changes in (**a**) fruit fresh weight; (**b**) fruit length, and (**c**) fruit diameter during fruit development of both paper-bagged and non-bagged 'Kurenainoyume' apples in 2018

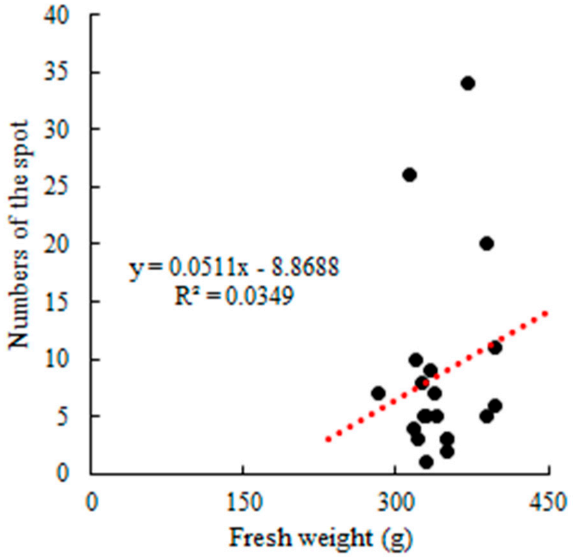

Figure S2. Relationship between the number of spots and fruit fresh weight of non-bagged 'Kurenainoyume' apples at harvest (October 23, 2019, 166 DAFB) (n = 20)
